# Supplementary material for: EnzML: multi-label prediction of enzyme classes using InterPro signatures
Source: BMC Bioinformatics. 2012 Apr 25;13:61. doi: 10.1186/1471-2105-13-61 (PMC3483700; doi:10.1186/1471-2105-13-61)
Supplement: Addtional file 5 — The Java code to format the data files, evaluate and predict. The file enzml_java_code.tar.gz contains the Java code used to format database data to ARFF and XML formats, to execute cross and train-test (jackknife) evaluations and to record evaluation results to database. More information is included in the readme.txt file and the Javadoc files. The code can be used with a MySQL database. To use a different database software, other JDBC drivers might be required. [file 1471-2105-13-61-S5.gz › java_code/utils/doc/cern/jet/random/AbstractContinousDistribution.html]

AbstractContinousDistribution


---


|  |  |  |
| --- | --- | --- |
| **Overview** \| **Package** \| **Class** \| **Use** \| **Tree** \| **Deprecated** \| **Index** \| **Help** | |  |
| PREV CLASS   **NEXT CLASS** | **FRAMES**    **NO FRAMES**     **All Classes** |  |
| SUMMARY: NESTED \| FIELD \| CONSTR \| METHOD | DETAIL: FIELD \| CONSTR \| METHOD |  |


---


## cern.jet.random Class AbstractContinousDistribution

```
java.lang.Object
  cern.colt.PersistentObject
      cern.jet.random.AbstractDistribution
          cern.jet.random.AbstractContinousDistribution
```

**All Implemented Interfaces:**: DoubleFunction, IntFunction, java.io.Serializable, java.lang.Cloneable

**Direct Known Subclasses:**: Pareto

---

``` public abstract class AbstractContinousDistribution extends AbstractDistribution ```

Abstract base class for all continous distributions.

**Version:**
:   1.0, 09/24/99

**Author:**
:   wolfgang.hoschek@cern.ch

**See Also:**: Serialized Form

---

**Method Summary**

**Methods inherited from class cern.jet.random.AbstractDistribution**

`apply, apply, clone, makeDefaultGenerator, nextDouble, nextInt`

**Methods inherited from class java.lang.Object**

`equals, getClass, hashCode, notify, notifyAll, toString, wait, wait, wait`

---


|  |  |  |
| --- | --- | --- |
| **Overview** \| **Package** \| **Class** \| **Use** \| **Tree** \| **Deprecated** \| **Index** \| **Help** | |  |
| PREV CLASS   **NEXT CLASS** | **FRAMES**    **NO FRAMES**     **All Classes** |  |
| SUMMARY: NESTED \| FIELD \| CONSTR \| METHOD | DETAIL: FIELD \| CONSTR \| METHOD |  |


---
